# The EYA Tyrosine Phosphatase Activity Is Pro-Angiogenic and Is Inhibited by Benzbromarone

**DOI:** 10.1371/journal.pone.0034806

**Published:** 2012-04-24

**Authors:** Emmanuel Tadjuidje, Tim Sen Wang, Ram Naresh Pandey, Saulius Sumanas, Richard A. Lang, Rashmi S. Hegde

**Affiliations:** 1 Division of Developmental Biology, Cincinnati Children's Hospital Medical Center, Cincinnati, Ohio, United States of America; 2 The Visual Systems Group, Division of Pediatric Ophthalmology, Cincinnati Children's Hospital Medical Center, Cincinnati, Ohio, United States of America; 3 Department of Pediatrics, University of Cincinnati, Cincinnati, Ohio, United States of America; 4 Department of Ophthalmology, University of Cincinnati, Cincinnati, Ohio, United States of America; Indiana University, United States of America

## Abstract

Eyes Absents (EYA) are multifunctional proteins best known for their role in organogenesis. There is accumulating evidence that overexpression of EYAs in breast and ovarian cancers, and in malignant peripheral nerve sheath tumors, correlates with tumor growth and increased metastasis. The EYA protein is both a transcriptional activator and a tyrosine phosphatase, and the tyrosine phosphatase activity promotes single cell motility of mammary epithelial cells. Since EYAs are expressed in vascular endothelial cells and cell motility is a critical feature of angiogenesis we investigated the role of EYAs in this process. Using RNA interference techniques we show that EYA3 depletion in human umbilical vein endothelial cells inhibits transwell migration as well as Matrigel-induced tube formation. To specifically query the role of the EYA tyrosine phosphatase activity we employed a chemical biology approach. Through an experimental screen the uricosuric agents Benzbromarone and Benzarone were found to be potent EYA inhibitors, and Benzarone in particular exhibited selectivity towards EYA versus a representative classical protein tyrosine phosphatase, PTP1B. These compounds inhibit the motility of mammary epithelial cells over-expressing EYA2 as well as the motility of endothelial cells. Furthermore, they attenuate tubulogenesis in matrigel and sprouting angiogenesis in the *ex vivo* aortic ring assay in a dose-dependent fashion. The anti-angiogenic effect of the inhibitors was also demonstrated *in vivo*, as treatment of zebrafish embryos led to significant and dose-dependent defects in the developing vasculature. Taken together our results demonstrate that the EYA tyrosine phosphatase activity is pro-angiogenic and that Benzbromarone and Benzarone are attractive candidates for repurposing as drugs for the treatment of cancer metastasis, tumor angiogenesis, and vasculopathies.

## Introduction

The Eyes Absent (EYA1-4) genes encode an unusual family of proteins; they have both transactivation and threonine phosphatase [1] activities in a poorly conserved N-terminal domain and tyrosine phosphatase activity in a well-conserved C-terminal domain [2], [3], [4]. The threonine phosphatase activity has been linked with the innate immune response, while the tyrosine phosphatase activity is associated with cell motility [5], DNA damage repair [6], [7], and fly eye development [2], [3]. Developmental defects encompassing multiple organs (including ear, kidney, muscle, thymus) have been reported in *Eya1* and *Eya4* mutant mice [8], and multi-organ birth defects are associated with *EYA* mutations in humans [9], [10], [11], [12]. In addition to the long-recognized developmental role of the EYAs, there is growing evidence that they are over-expressed in various adult cancers; EYA2 (in breast [5], [13] and ovarian cancer [14]) and EYA4 (in malignant peripheral nerve sheath tumors [15]) are associated with increased tumor size and metastasis. The tyrosine phosphatase activity specifically promotes the motility and invasiveness of cancer cells [16].

Evidence for a link between the EYAs and cardiovascular development has been growing; mutation of *EYA4* is associated with dilated cardiomyopathy [17], human *EYA1* mutations are associated with cardiac defects [18], there are alterations in cardiovascular function in *Eya3* mutant mice [19], and Six1^-/-^Eya1^-/-^ mutant mice exhibit multiple vascular anomalies [20]. However a direct link between EYA and the process of angiogenesis has not been reported. Moreover, because the EYAs have multiple biochemical activities, the molecular mechanisms by which EYA might influence vascular development remains to be established. Chemical biology has emerged as an effective means of dissecting the cellular and biological roles of such multifunctional proteins. However, achieving specificity among protein tyrosine phosphatases has been a challenge for inhibitor design. In this regard the EYAs could have an advantage since the EYA tyrosine phosphatase domain is mechanistically unusual; it does not utilize a cysteine residue in catalysis, as do all other known protein tyrosine phosphatases (PTPs) (reviewed in [21]). Instead, the EYAs belong to the large family of haloacid dehalogenase (HAD) type phosphotransferases, metallo-enzymes that employ an Aspartate as a nucleophile and another conserved Aspartate two residues downstream as an acid catalyst [2], [22]. The active site of the EYAs thus represents an unconventional target for the design of small molecule PTP inhibitors.

**Figure 1:**
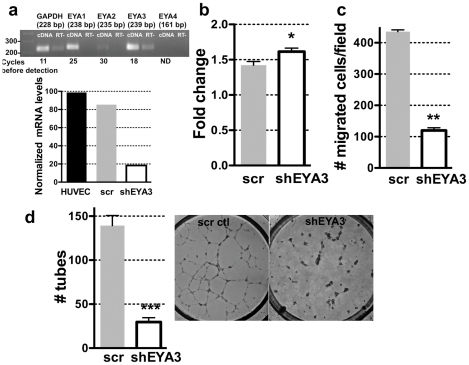
EYA3 knockdown in HUVECs attenuates single cell motility and capillary morphogenesis. (**a**) Expression of Eya transcripts in HUVECs. Real-time PCR product was analyzed at saturation (cycle 45) on a 1.5% agarose gel to confirm that amplified products were of the expected sizes. Indicated are the numbers of amplification cycles after which each signal was detectable; EYA4 was not detected (ND) after 45 cycles of amplification. RT- stands for cDNA from a reaction without reverse transcriptase. Graph below show the results of quantitative RT-PCR on HUVECs transduced with lentivirus scramble control RNA or shEYA3. (**b**) Change in cell density of HUVECs-scramble control and HUVEC-shEYA3 after 24 hours shows a small but significant increase when EYA3 levels are reduced. (**c**) Transwell migration of HUVEC-scramble control and HUVEC-shEYA3 shows a significant reduction in motility when EYA3 levels are reduced. (**d**) Capillary morphogenesis on matrigel was assayed using HUVEC-scramble control and HUVEC-shEYA3. A significant reduction in tube formation was seen when EYA3 levels were lowered. In each case the bars represent the mean and standard error of three experiments. The *p*-values were calculated by one-way ANOVA followed by a Tukey post test. ns is not significant, * *p*<0.05, ** *p*<0.01, *** *p*<0.001. In each case the p value shown is relative to the scramble-control.

Here we show that the uricosuric agents Benzbromarone and Benzarone are potent inhibitors of the EYA tyrosine phosphatase. Both compounds exhibit selectivity for EYA over a representative classical PTP, PTP1B. They are active in cellular assays, inhibiting the EYA-promoted motility of mammary epithelial cells as well as endothelial cells. Furthermore we provide evidence that inhibition of EYA attenuates angiogenesis using *in vitro, ex vivo* and *in vivo* assays. This is the first direct demonstration of a role for the EYA proteins in angiogenesis and raises the possibility that inhibition of EYA activity could be a useful therapeutic strategy in the treatment of tumor angiogenesis and other vasculopathies. Additionally, since EYA over-expression and catalytic activity is linked to tumor metastasis and DNA damage repair, an inhibitor of the EYA tyrosine phosphatase could be useful as an anti-metastatic agent and in the potentiation of DNA damaging therapies.

## Results

### Eyes Absents are expressed in endothelial cells and contribute to endothelial cell migration and capillary tubule formation

Eyes absent (EYA1) plays an important role in pulmonary and cardiac vascular morphogenesis. [23], [24]. Prior to investigating a cell-autonomous requirement of Eya in endothelial cells function, RT-PCR was performed in order to identify which Eya transcripts are expressed in vascular endothelial cells. mRNA for EYA1 and EYA3 was strongly detected in human umbilical vein endothelial cells (HUVECs) (Figure 1a). To evaluate the role of EYA in endothelial cell function HUVECs were stably infected with lentiviruses expressing either control shRNA (scramble control) or an shRNA specific to EYA3. Quantitative real-time PCR (qRT-PCR) was used to estimate the reduction of mRNA level relative to the scramble control and showed nearly 75% reduction in transcript level (Figure 1a). To assess the effect of reducing EYA3 expression on cell proliferation the colorimetric tetrazolium salt MTT assay [25], which monitors the metabolic activity of cultured cells, was used. HUVEC-shEYA3 cells showed a small but significant increase in proliferation compared to control cells (Figure 1b). Next a modified Boyden chamber assay was conducted and a significant attenuation of single cell migration upon knockdown of EYA3 was detected (Figure 1c). Since cell migration is critical for angiogenesis, we next examined the effect of EYA3 levels on the morphological differentiation of HUVECs in Matrigel. Under control conditions HUVECs formed a network of capillary-like structures in 20 hours while HUVECs in which EYA3 levels were reduced were severely compromised in their ability to form tubes (Figure 1d). Together these data support a role for EYA3 in endothelial cell remodeling, most likely by promoting cell migration.

### Identification of a small molecule inhibitor of the EYA tyrosine phosphatase

**Figure 2:**
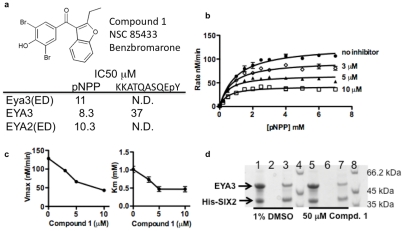
EYA inhibitor Benzbromarone, compound 1. (**a**) Compound 1 was identified from a screen of the NCI Diversity Set II library. IC50 values for compound 1 were measured for Eya3(ED), human EYA3 and human EYA2(ED) using as substrates the phosphotyrosine mimic pNPP and a phosphopeptide representing the last 10 amino acids of H2AX, a known EYA substrate. (**b**) Substrate titration shows that compound 1 is an uncompetitive inhibitor of EYA3(ED). Increasing concentration of substrate does not overcome inhibition. Each point represents the mean and standard deviation of two independent readings. (**c**) Plots of Vmax and Km as a function of inhibitor concentration show that both values decrease with increased inhibitor concentration. Values in (c) were derived from nonlinear regression analyses of curves in (b) using PRISM (GraphPad Software). (**d**) Compound 1 does not affect the interaction between EYA3 and SIX2. Recombinant purified EYA3 and His-SIX2 were mixed and treated with either the vehicle control (1% DMSO) (lane 1) or 50 μM compound 1 (lane 5) for 15 minutes at room temperature. The mixture was loaded on a Ni-NTA column. Beads were washed with 3 column volumes of load buffer (last washes, lanes 2 and 6). Proteins retained on the beads are shown in lanes 3 and 7. Lanes 4 and 8 are molecular weight markers.

A specific chemical inhibitor of the EYA tyrosine phosphatase activity represents a convenient means of querying the role of this activity in cell motility and angiogenesis while leaving the transactivation and threonine phosphatase domains unaffected. Towards this goal we conducted an experimental screen to identify chemical inhibitors. The National Cancer Institute's Diversity Set II Library was screened for inhibitors of the phosphatase activity of Eya3. Hydrolysis of the model substrate p-nitrophenylphosphate (pNPP) by the catalytic domain of Eya3 (ED; residues 223–510) in the presence of 125 µM of each compound was measured. The 30 strongest inhibitors were then tested using full-length human recombinant, purified EYA3 and pNPP as a substrate. These screens led to the identification of Benzbromarone (compound 1; Figure 2) as a potent inhibitor of EYA3's tyrosine phosphatase activity. Compound 1 also inhibited the catalytic activity of EYA2(ED) with comparable potency. These results were mirrored when an alternate substrate, a 10 amino acid phosphopeptide representing the C-terminus of the known EYA substrate γ-H2AX [6], [7], was used (Figure 2). The inhibitory effect was retained in the presence of 0.01% Triton X-100, a non-ionic detergent, indicating that compound 1 was not non-specifically self-aggregating and sequestering enzyme as has been observed in some cases [26], [27].

The catalytic domain of the EYAs also mediates its interaction with the SIX proteins. This complex then translocates to the nucleus where the SIX-EYA complex can activate transcription [28]. To determine whether this series of compounds might disrupt a representative EYA-SIX interaction we tested the ability of His-tagged SIX2 to pull-down (using Ni-NTA agarose) EYA3 in the presence and absence of compound 1. The interaction appeared to be unaffected by the presence of the EYA inhibitor (Figure 2d).

**Figure 3:**
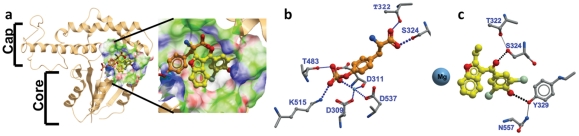
Binding modes of Benzbromarone and the substrate mimic phosphotyrosine differ. (**a**) In docking experiments phosphotyrosine (gold) and compound 1 (yellow) bind at the interface of the cap and core subdomains of the catalytic ED domain of EYA3. (**b**) Phosphotyrosine coordinates the active site metal ion and makes hydrogen bonds with residues in all three conserved motifs in the EYAs. (**c**) Docked conformation (ICM Score −24.8) of compound 1 docked into the active site of EYA3(ED). The conformation shown here was obtained in multiple docking experiments conducted with different starting conformations for the ligand. Compound 1 inserts its dibromo-phenol group into a hydrophobic cavity adjacent to the phosphotyrosine binding site and is anchored by a hydrogen bond with Tyr 329. The carbonyl group forms a hydrogen bond with Ser 324. Compound 1 is not within bonding distance of the metal ion.

To gain a better understanding of how EYA3 might bind compound 1, we generated a homology model of EYA3(ED) using the crystal structure of EYA2(ED) bound to BeF_3_ (3HBO.PDB, [7], [29]) as a template and the SWISSMODEL server [30]. Structure-based alignment of residues 302–573 of EYA3 with residues 267–568 of EYA2(ED) was used. Residues 356–370 of Eya2 are not present in the Eya2(ED) crystal structure, presumably because they are disordered. The corresponding region in EYA3 (391–405) is omitted from the final model. The proteins share 67% sequence identity. The resulting homology model was validated using ANOLEA [31], VERIFY-3D and GROMOS [30]. These analyses indicated that the molecular geometry of the model was of good quality. The structure of EYA3(ED) is comprised of two subdomains: residues 316–458 form the “cap” domain while the rest of the residues make up the “core” catalytic domain (Figure 3a). The active site (represented by the Mg^2+^ ion in Figure 3a) is at the interface of the two subdomains.

In order to provide a benchmark for modeling the interaction between EYA and the inhibitors identified here, phospho-tyrosine was docked into the active site of EYA3(ED) using ICM-Chemist-Pro developed by Molsoft [32]. The docking was performed with multiple starting locations for the ligand. Since the EYAs dephosphorylate the C-terminal phosphotyrosine of γ-H2AX [6], [7] a free backbone carboxylate was used. The docked conformation for the phosphotyrosine ligand is shown in Figures 3a, b. Non-esterified phosphate oxygens of phosphotyrosine coordinate the Mg^2+^ ion (2.1 Å) and are also within hydrogen bonding distance of the side-chains for Lys515 and Thr483. The free carboxylate of the tyrosine residue is positioned to form hydrogen bonds with side-chains of Thr 322 and Ser 324 at the interface of the cap and core domains; such substrate anchoring by HAD family phosphotransferases that have a cap domain is commonly observed. Residues 515, 483, 322 are identical between the EYAs, while Ser324 is a Thr in other EYA proteins. Lys515 is in motif III as originally described by analogy with other haloacid dehalogenase class proteins and Thr483 is adjacent to the conserved Thr in motif II [2]. Thus, this mode of phosphotyrosine binding agrees with predictions made by analogy with other haloacid dehalogenase enzymes and is likely to be shared among the EYA proteins.

Next, compound 1 was docked into the active site of EYA3(ED). The results are shown in Figure 3c. The dibromophenol moiety inserts into a pocket adjacent to the active-site (as defined by the divalent metal ion, the nucleophilic Asp309 and the general acid Asp311), and at the interface of the cap and core domains. This pocket is lined by the side-chains of residues Glu 312, Thr 313, Ile 316, Ser 324, Tyr 325, Lys 328, Tyr 329, and the loop connecting the C-terminal β-strand and helix of the ED domain. Hydrogen bonds to Tyr329 and Ser324 tether the dibromophenol in site I. Ser 324 also plays a role in phosphotyrosine binding but, as is apparent from Figure 3a, there is little other similarity between the modes of pY and inhibitor binding. Notably compound 1 does not appear to chelate the metal ion or occupy the phosphate-binding site predicted by the docking of phosphotyrosine in Figure 3 or by the binding of BeF_3_ and AlF_3_ in the published crystal structures [29]. This suggests that compound 1 may not compete with phospho-tyrosine for the same site. To test this prediction the velocity of pNPP (a phosphotyrosine mimic) hydrolysis by Eya3(ED) in the presence and absence of a range of concentrations of compound 1 was measured. Figures 2b and 2c show that Eya3(ED) inhibition by compound 1 was not overcome by increasing substrate concentrations and is best described by an uncompetitive inhibition model.

### Structure-activity relationships of Compound 1

**Figure 4:**
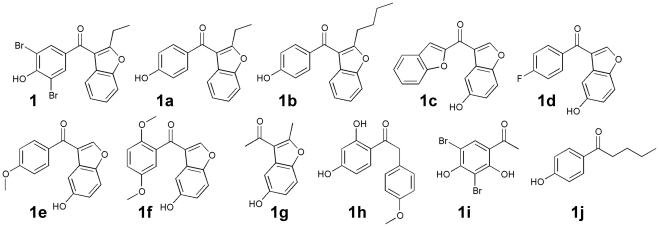
Structure-activity relationships. Chemical structures of compounds related to Benzbromarone (1a–1j) that were assayed for their ability to inhibit the phosphatase activity of Eya3(ED), EYA3, and PTP1B. IC50 values are in Table 1.

A set of 10 structurally related compounds (Figure 4) were purchased and screened for their ability to inhibit the catalytic activity of Eya3(ED) and full-length EYA3. The results are shown in Table 1. Compounds 1a and 1b, which retain the basic scaffold of a phenol and a benzofuran linked by a carbonyl group, had IC50 values comparable to those of compound 1. Deletion of the two bromine atoms or increasing the length of the aliphatic substituent on the benzofuran did not significantly affect the inhibition. However compounds 1c–1g which lack the phenol and 1h–1j which lack the benzofuran group were significantly worse inhibitors, suggesting that both moieties are necessary for an active EYA inhibitor.

### EYA inhibitors attenuate cell migration but not cell proliferation

**Figure 5:**
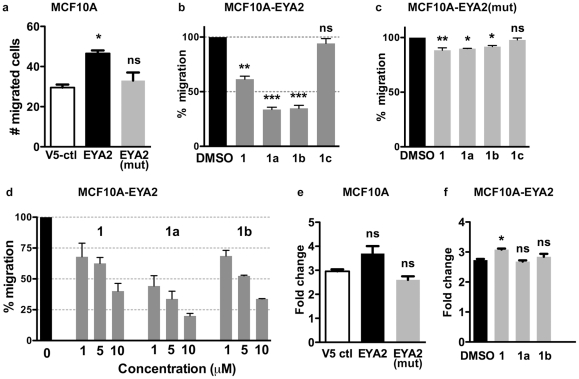
EYA2 overexpression in MCF10A cells increases cell motility, which in turn is inhibited by EYA inhibitors. (**a**) Trans-well migration of MCF10A cells transfected with either pcDNA 3.2/V5-DEST (V5 vector control), V5-EYA2, or the phosphatase dead mutant V5-EYA2(mut). (**b**) MCF10A-EYA2 cells were treated with 7.5 μM of compounds 1, 1a, 1b, or 1c. Percentage migration relative to cells treated with the vehicle (0.1% DMSO) is shown. (**c**) Transwell migration of MCF10A-EYA2(mut) cells in the presence of 7.5 μM of compounds 1, 1a, 1b, or 1c (**d**) Relative migration of MCF10A-EYA2 cell migration by compounds 1, 1a, and 1b when treated with the doses indicated on the x-axis. (**e**) Change in cell density of MCF10A-V5 ctl, MCF10A-EYA2 and MCF10A-EYA2(mut) cells after 48 hours measured using the MTT assay. (**f**) Change in cell density of MCF10A-EYA2 cells after 48 hours in the presence of either vehicle control (0.1% DMSO) or 10 μM compounds 1, 1a, and 1b. For transwell migration experiments each bar represents the mean (and standard error) of five random fields per filter and two wells per experiment. For proliferation experiments each bar represents the mean (and standard error) of three experiments. The *p*-values were calculated by one-way ANOVA followed by a Tukey post test. ns is not significant, * *p*<0.05, ** *p*<0.01, *** *p*<0.001. In each case the *p* value shown is relative to the V5-control or the vehicle-treated sample.

In previous studies we have shown that the tyrosine phosphatase activity of Eya3 and Eya2 promotes single cell motility in breast cancer cells [16]. EYA2 is over-expressed in breast cancers and is associated with increased metastasis and a poorer outcome [13]. In order to determine whether the EYA inhibitors were effective in an established EYA tyrosine-phosphatase-associated cellular function, we over-expressed either EYA2 or EYA2(mut) (in which the nucleophilic Asp is replaced by an Asn rendering the enzyme inactive) in the immortalized, non-transformed mammary epithelial cell line MCF10A. Transwell migration was measured and, as anticipated, based on previous studies [16], over-expression of EYA2, but not the tyrosine-phosphatase dead mutant, promoted cell motility (Fig. 4a). Next the migration of MCF10A(EYA2) cells in the presence of 7.5 μM of compounds 1, 1a, 1b, and 1c was measured (Figure 5b). All of them, except 1c, significantly inhibited cell migration. This effect was dose-dependent (Figure 5d) and the most effective compound (1a) was able to reduce cell motility by over 50% at the lowest concentration (1 μM) tested. Notably, compound 1c, which inhibits PTP1B but not EYA, had no effect on cell motility. The effect of the EYA inhibitors on the motility of cells over-expressing the phosphatase-dead mutant EYA2(mut) was comparable to that observed with control MCF10A cells (Figure 5c).

**Table 1:** 

	IC50 μM (pNPP)		
COMPOUND	Eya3(ED)	EYA3	PTP1B
1 Benzbromarone	11	8.3	53.8
1a Benzarone	19.2	17.0	>150
1b	10.1	15.2	71.4
1c	>150	>150	11.8
1d	>150	>150	>150
1e	>150	>150	>150
1f	>150	>150	102.1
1g	>150	>150	>150
1h	>150	>150	>150
1i	>150	>150	>150
1j	>150	>150	>150

This same series of compounds was assayed for their effect on pNPP hydrolysis catalyzed by the classical Cys-based tyrosine phosphatase PTP1B. Compounds 1 and 1b showed 4.7–6.5 fold greater activity towards EYA3 than PTP1B, while compound 1a was over 100-fold more specific towards EYA3. On the other hand compound 1c preferentially inhibited PTP1B.

To assess the cellular toxicity of the compounds and their effect on cell proliferation, an MTT assay was conducted. Over-expression of EYA2 or the mutant EYA2(mut) had insignificant effect on MCF10A cell proliferation (Fig. 5e), and none of the EYA inhibitors led to any significant change in proliferation of MCF10A-EYA2 cells (Figure 4f). Together these data suggest that the EYA tyrosine phosphatase inhibitors 1, 1a and 1b can reduce the motility of mammary epithelial cells over-expressing EYA2 at concentrations that are not cytotoxic.

### EYA tyrosine phosphatase inhibitors attenuate endothelial cell motility and tubulogenesis in vitro

**Figure 6:**
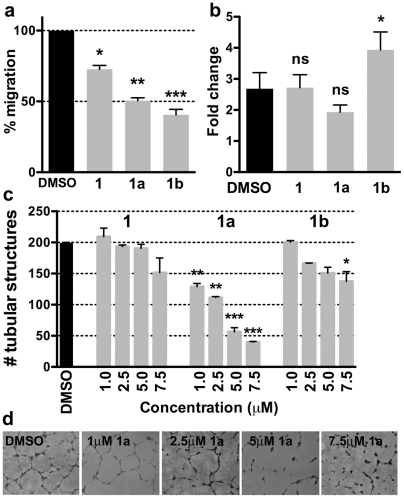
EYA inhibitors attenuate migration and tubulogenesis of HUVECs. (**a**) Percent migration of HUVECs in the presence of 5 μM relative to the vehicle control?. (**b**) Change in cell density after 24 hours in the presence of either vehicle control (0.1% DMSO) or 5 μM of each EYA inhibitor. (**c**) Quantitation of the number of tube-like structures formed by HUVECs in the presence of either the vehicle control (0.1% DMSO) or the indicated concentrations of compounds 1, 1a and 1b. The number of tubes was measured using NeuroJ. Data are mean and standard error of three independent experiments. *p-values* from a one-way ANOVA are shown; ns is not significant, * *p*<0.05, ** *p*<0.01, *** *p*<0.001. (**d**) Representative images of HUVECs on Matrigel in the presence of the indicated doses of 1a.

We next sought to specifically query the role of the EYA tyrosine phosphatase activity in endothelial cell migration and angiogenesis. HUVECs in the presence of compounds 1, 1a, 1b, 1c, or a vehicle control (0.1% DMSO) were used in transwell migration assays. As in the case of MCF10A-EYA2 cells, all of these compounds (except 1c) attenuated HUVEC cell motility (Figure 6a). In parallel experiments using MTT no reduction of cell proliferation was observed (Figure 6b). The EYA inhibitors were also used in tubulogenesis assays conducted on Matrigel. Compound 1a potently inhibited tubulogenesis in a dose-dependent fashion, showing nearly 50% reduction in tube formation at 2.5 μM (Figure 6c). Compounds 1 and 1b were effective only at the highest concentrations tested (7.5 μM). Thus the EYA inhibitors are able to reduce endothelial cell motility and tube formation.

### EYA inhibitors inhibit sprouting angiogenesis in an ex vivo murine aortic ring assay

**Figure 7:**
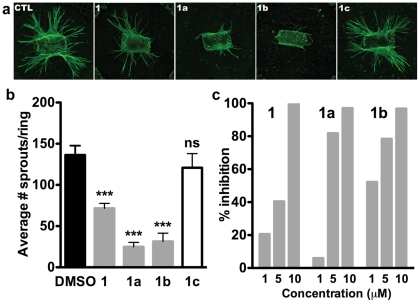
EYA inhibitors attenuate sprouting angiogenesis. (**a**) Representative images of aortic rings treated with either the vector control (0.1% DMSO) or 5 μM of compounds 1, 1a, or 1b. Rings were stained with isolectin. 1c is used as a negative control as it does not inhibit EYA. (**b**) Quantitation of the number of sprouts per ring is included; each bar represents the mean of 6 rings. *p-values* from a one-way ANOVA are shown; ns is not significant, * *p*<0.05, ** *p*<0.01, *** *p*<0.001. (**c**) Compounds 1, 1a, and 1b in the indicated doses were used in aortic ring experiments. The number of sprouts per ring is plotted indicating that inhibition of aortic sprouting was dose-dependent.

To further examine the role of the tyrosine phosphatase activity and the effectiveness of the inhibitors on vascular maturation and morphogenesis we used the murine aortic ring assay. In this protocol angiogenesis is monitored by the formation of vascular sprouts outside the wall of mouse aortic rings incubated in collagen matrix and stimulated by VEGF [33]. An extensive microvascular network with an average maximal sprout length of 900 μm was observed in both untreated and vehicle-treated (0.1% DMSO) mouse aortic rings after 10 days (Figure 7a). In contrast, aortic rings treated with 5 μM of compounds 1, 1a, and 1b exhibited shorter sprouts and significantly lower microvascular density (Figure 7a). Moreover this inhibition was dose-dependent (Figure 7c) and compound 1c had negligible effect. These results demonstrate that inhibition of EYA tyrosine phosphatase activity reduces the angiogenic response to VEGF stimulation.

### EYA tyrosine phosphatase inhibition has an anti-angiogenic effect in a zebrafish model

**Figure 8:**
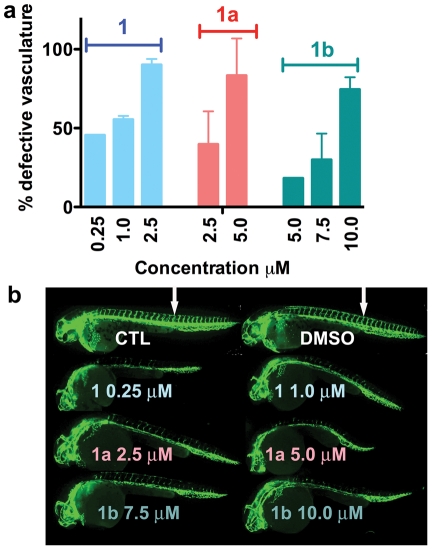
Dose-dependent effects of EYA inhibitors on the developing zebrafish vasculature. (**a**) Titration of compounds 1, 1a, 1b at the indicated doses. Two independent experiments were performed in most cases using 9–15 embryos per experiment at each dose; the standard error and mean values are shown. Experiments with 0.25 μM compound 1 and 5 μM compound 1b were only performed once using 10 embryos each. The y-axis shows the percentage of embryos showing any defects in vascular development (either intersegmental vessels (ISV) formation or defects in the dorsal aorta/cardinal vein). (**b**) Images of representative untreated control (CTL), vehicle (DMSO) treated, and EYA inhibitor treated embryos at 28–30 hpf. Note reduced or absent ISV in the inhibitor treated embryos relative to the controls (ISV in controls indicated by white arrows).

In order to test the possibility that the EYAs are angiogenic in the more complex, context-driven environment encountered by blood vessels *in vivo* we used the well-characterized zebrafish model of angiogenesis [34], [35]. Transgenic fish expressing EGFP in endothelial cells (*Tg(kdrl:EGFP)*) [36] were used to facilitate visualization of the developing vasculature. In control experiments embryos were treated with 0.1% DMSO (vehicle) starting at approximately 5 hours post-fertilization (hpf), prior to the emergence of blood vessels. At the time of analyses (28–30 hpf) the major axial vessels, the dorsal aorta, the posterior cardinal vein and intersegmental vessels which branch from the dorsal aorta in a stereotypical pattern were normally formed (Figure 8b). Experimental embryos were exposed to varying doses of compounds 1, 1a and 1b. Dose-dependent defects in the developing vasculature were observed in all cases (Figure 8a), ranging from a reduction in intersegmental vessel number and extension at lower doses to changes in the dorsal aorta and cardinal vein at higher doses. Representative images are shown in Figure 8b. Compound 1 (Benzbromarone) was the most potent showing significant reduction of intersegmental vessels even at the lowest dose tested (0.25 μM). Benzarone (compound 1a) and 1b also reduced the number of intersegmental vessels, but were less potent. When embryos were exposed to the compounds starting at a later stage (20 hpf) no defects in the vasculature or general morphology were observed. Compound 1c, which does not inhibit the EYAs but does inhibit PTP1B, was used as a negative control; at the highest dose tested (7.5 μM) it showed no effect on the vasculature. This analysis confirms that Eya plays a role in promoting developmental angiogenesis and that the EYA inhibitors are effective in an *in vivo* experimental system.

## Discussion

In this report we demonstrate that Eyes Absent promotes angiogenesis specifically through its tyrosine phosphatase activity. While a role for the EYAs in angiogenesis has not been previously reported, it is not an unexpected observation in light of the association between the EYAs and cell migration, and the cardiovascular defects associated with Eya mutations in humans and in mice. Effects of EYA inhibition were apparent in the processes of both vasculogenesis (HUVEC capillary tube formation, formation of the zebrafish aorta and posterior cardinal vein) and sprouting angiogenesis (aortic ring assay, formation on intesegmental vessels in zebrafish). Upon bathing zebrafish embryos in the EYA inhibitors we see a clear effect on vascular development at doses that do not affect the general morphology, suggesting a degree of specificity. On the other hand when the inhibitors were administered relatively late no significant change in the development of intersegmental vessels was seen. Hence from the currently available data it is not possible to specify whether the inhibitors act exclusively on either vasculogenesis or angiogenesis. *In vitro* EYA inhibition does not appear to attenuate endothelial cell proliferation. Hence the pro-angiogenic function of EYA could be a direct result of its ability to activate a cell migration-promoting pathway. The EYA substrate involved in this process is yet to be identified.

However another possibility is that dephosphorylation of H2AX at Tyr142 [6], [7] underlies the pro-angiogenic role played by EYA. Angiogenesis can be promoted by hypoxia during normal development as well as in pathological conditions such as retinopathy of prematurity (ROP), diabetic retinopathy and tumor angiogenesis (reviewed in [37]). Hypoxia leads to DNA damage and the phosphorylation of H2AX at Ser139 (to yield γ-H2AX) by ATR kinases. Re-oxygenation and the production of reactive oxygen species lead to double-strand breaks and the formation of γ-H2AX though the action of ATM kinase. Recent evidence suggests that such activation of H2AX contributes to angiogenesis and proliferative retinopathies[38]. The constitutively Tyr142-phosphorylated form of H2AX [39] promotes recruitment of the p53 apoptosis machinery and disfavors DNA damage repair [6]. Activation of ATM/ATR also leads to EYA activation and dephosphorylation of H2AX-pY_142_, thus permitting DNA repair [6], [7]. Hence hypoxia-induced angiogenesis could be promoted by the tyrosine phosphatase activity of the EYA proteins via dephosphorylation of H2AX-pY_142_. In this manner EYA is likely to play a role in both developmental and pathological retinal angiogenesis.

In this study small molecule inhibitors of the Eyes Absent tyrosine phosphatase activity were used as chemical probes of EYA's cellular function because they permit separation of EYA's signal transduction and transcriptional activities. However the inhibitors also have other potential uses. The growing evidence that EYAs are over-expressed in malignancies raises the possibility that inhibitors could be a targeted therapy for appropriate subsets of breast and ovarian cancers, as well as malignant peripheral nerve sheath tumors [13], [14], [15], [16]. Furthermore, since the EYA PTP activity promotes DNA damage repair [7], it is likely that EYA inhibitors could potentiate commonly used genotoxic therapeutic regimens such as chemotherapy and ionizing radiation.

Interestingly Benzbromarone was also identified in an early screen for PTP1B inhibitors [40], and more detailed analyses and crystallographic studies of Benzbromarone-derivatives were later reported [41]. These compounds bind to an allosteric site on PTP1B far from the active site and are noncompetitive inhibitors that prevent formation of the active “closed” conformation of the enzyme. In contrast our studies suggest that Benzbromarone binds in a hydrophobic pocket adjacent to the active site of EYA, and it is not competitive with small substrate mimics such as pNPP and phosphotyrosine. Furthermore both the docking studies (Figure 3) and kinetic analyses (Figure 2) demonstrate that, despite the presence of a phenol group in all of the EYA inhibitory compounds in Figure 4, they are unlikely to be acting as simple tyrosine-mimics. Inhibition of both EYA3 and EYA2 were tested in this analysis and the compounds are similarly effective. This result is not surprising given the high degree of sequence conservation in the predicted inhibitor binding sites. Analyses of benzbromarone related compounds in Figure 4 has also permitted a separation of the chemical scaffolds necessary for EYA inhibition versus PTP1B inhibition; compound 1a is preferentially an EYA inhibitor while compound 1c is a PTP1B inhibitor. These data thus provide a basis for the design of more potent and selective EYA inhibitors.

EYA is a metallo-enzyme [2], [22], [42]. However the inhibitors described here are not metal-chelators, unlike compounds identified in a recently reported *in silico* screen performed using the isolated catalytic domain of EYA2 [43]. While metal chelators can be high affinity inhibitors of metalloenzymes, as therapeutics they present challenges such as depletion of physiologically needed metal ions and inhibition of non-targeted metalloenzymes. Hence the identification of EYA inhibitors that do not appear to coordinate the active site divalent metal in the EYAs opens up new possibilities.

Compound 1 (Benzbromarone) and compound 1a (Benzarone) are both known uricosuric agents. They suppresses uric acid reabsorption via inhibition of the urate transporter SLC22A12 (URAT1) [44]. The compounds have a short half-life (about 3 hours [45]), but their major metabolite 6-hydroxybenzbromarone has a longer half-life of 30 hours and retains the uricosuric activity. These compounds are no longer marketed for use in the treatment of gout in most countries because of reported hepatotoxicity and their ability to inhibit CYP2C9 which causes undesired interactions with drugs that use this enzyme for clearance, such as warfarin (reviewed in [46]). However there remains controversy about whether the benefit-risk ratio warrants a complete withdrawal [47]. There have been no previous reports of either compound affecting cell motility or angiogenesis. Hence the data presented here represents an opportunity for re-purposing a well-characterized drug as an anti-angiogenic and anti-metastatic agent.

In summary we have identified a class of compounds that strongly inhibit the EYA tyrosine phosphatases. These compounds display selectivity towards EYA relative to a representative classical PTP, PTP1B. The inhibitors effectively attenuate the known ability of the EYAs to promote cell motility and have been used as a tool to reveal a pro-angiogenic function for the EYA tyrosine phosphatase. These inhibitors could thus be useful in identifying other biological role(s) of this multi-tasking protein, as well as in probing the molecular mechanisms by which it functions. The compounds also represent viable leads for the development of EYA-targeted therapeutics with potential uses in the treatment of cancer metastasis, potentiating DNA damaging chemotherapy, tumor angiogenesis, as well as other vasculopathies.

## Materials and Methods

### Ethics Statement

All animal use and experimentation was in accordance with protocols approved by Cincinnati Children's Hospital Medical Center's Institutional Animal Care and Use Committee. 4–6 week old C57BL/6 mice were obtained from the Jackson Labs (Bar Harbor, Maine) and housed in the Cincinnati Children's Hospital Animal Facility.

### shRNA mediated mRNA depletion

HUVECs were incubated overnight with shEYA3 or scramble control lentiviral suspension in the presence of 8 μg/ml polybrene. The next day, viral suspension was replaced by fresh medium. 24 hrs later, cells were selected with 2 μg/ml puromycin until control cells were all dead (after 72 hrs of selection). To measure expression of Eya transcripts in HUVECs total RNA was extracted from 1000,000 HUVECs, cDNA was synthesized from 2 µg of total RNA using the Superscript III kit (Invitrogen) in a total volume of 30 μl. cDNA was diluted with nuclease free water to 180 μl. 5 μl of the diluted cDNA was used for real-time PCR. PCR product was analyzed at saturation (cycle 45) on a 1.5% agarose gel to confirm that amplified products were of the expected sizes. Indicated are the numbers of amplification cycles after which each signal was detectable; EYA4 was not detected (ND) after 45 cycles of amplification. RT- stands for cDNA from a reaction without reverse transcriptase. Primers used are listed below.

hEYA1: CACGATGCTAATCCGGAAAT and AATATTGCCCAGCCAAACTG

Location on cDNA: 3322–3459

hEYA2: GATTCGGCAGAAAAGCTGTC and CTGGGTCTCTGGAGTTCTCG; Location on cDNA: 1606–1840

hEYA3: CACGCCTGGCTAATTTTTGT and CCTCAACCCAAGACACACCT; Location on cDNA: 4913–5151

hEYA4: TGCTAAGTCTGTGGGTGCAG and GTTCTCCGTCAACCTTGCAT; Location on cDNA: 4666–4826

GAPDH: CGACCACTTTGTCAAGCTCA and AGGGGTCTACATGGCAACTG; Location on cDNA: 964–1191

### Transwell migration assays

Human umbilical vein endothelial cells (HUVECs) were purchased from Lonza (Walkersville, MD) and maintained in the endothelial cell growth medium (PromoCell, Heidelberg, Germany), under a 5% CO2 atmosphere. MCF10A cells from ATCC were stably transfected with either pcDNA 3.2/V5-DEST (vector control), V5-EYA2 or V5-EYA2(mut). Transwell migration experiments were performed as previously described [16].

### Cell proliferation assay

HUVEC cells were plated at 2,000 cells/100 μl/well in a collagen I coated 96-well plate and cultured at 37°C in a humidified incubator in the presence of 5% CO_2_. For each condition and time point, the culture was set up in triplicate. After the desired incubation time, the number of viable cells was estimated using the cell counting kit-8 (Dojindo Molecular Technologies, Rockville, MD). The cell density was expressed as the mean absorbance at 450 nm.

### Tubulogenesis assay

Tubulogenesis assays were performed in 15-well micro-slide (ibidi LLC, Verona WI), using growth factor-reduced matrigel (BD Bioscience, Billerica, MA). The matrix was prepared by loading 10 μl of matrigel in each micro-slide well and allowing it to solidify for 30 minutes at 37°C. HUVECs were trypsinized and resuspended at 100,000 cell/ml in EBM +2.5% FBS. 50 μl (5,000 cell) were loaded on top of the solidified matrigel and the preparation was incubated for 20 hours at 37°C in a humidified incubator in a 5% CO_2_ atmosphere. Bright field images were taken using an inverted microscope at 2.5× magnification, which allowed imaging of the whole well in two pictures that were later merged in Photoshop. Tubular structures were traced and counted using NeuroJ (NIH, USA). In experiments with inhibitor, the inhibitor was added to the cell suspension before loading. Each sample was loaded in triplicate, and each treatment was repeated for reproducibility.

### Purification of recombinant proteins

The catalytic domain (ED) of mouse Eyes Absent 3 (Eya3(223–510)) was purified as described before [42]. Human Eyes Absent 3 isoform 2 (representing the major species identified in human cell lines by mass spectrometry [48] and residues 127–573 of NM_001990, isoform 1) was sub-cloned into pDEST565 to express a poly-histidine- Glutathione-S-transferase (His-GST) tagged fusion protein with a TVMV protease site. The protein was purified by glutathione-S-transferase affinity chromatography followed by TVMV cleavage, Ni-NTA chromatography, and finally size exclusion chromatography.

The catalytic domain of human Eyes Absent 2 was similarly sub-cloned as a poly-histidine fusion construct in the vector pDEST-527. Fusion protein was purified by Ni-NTA chromatography and followed by size-exclusion chromatography over a Superdex-75 column. The relative purity of protein samples was determined using SDS-PAGE. PTP1B was purified as described previously [2].

### Inhibitor Screen

The NCI Diversity Set II was obtained from the NCI in 96-well trays from the NIH Molecular Libraries Program and stored as 10 mM stocks in DMSO. An initial manual screen was conducted using the previously described p-nitrophenylphosphate assay [2] and mouse Eya3(223–510) in a reaction mixture containing 20 mM MES pH 6, 2 mM MgCl_2,_ 125 μM inhibitor, 3.4 mM para-nitrophenol phosphate (pNPP) and 0.01 μg/µL enzyme. The amount of 4-nitrophenol (pNP) produced was monitored at 405 nM on a BioTek EL808 plate reader. A similar protocol was used to screen for inhibitor activity using hEYA3 and hEYA2(ED).

### IC_50_ determination

Compound 1a was obtained from Sigma-Aldrich (cat. no. L129305) and compounds 1b–1j from ChemDiv (cat. nos. 3039-0682; 4237-0212; 6526-0043; 6526-0210; 8005-2108; 8009-4552; 0831-0939; 8005-2010; R052-0049). Compounds were dissolved in DMSO and diluted as needed. IC_50_ values were determined by adding varying amounts of inhibitor (0–400 μM) to reaction mixtures containing 20 mM MES pH 6, 2 mM MgCl_2,_ 2% DMSO, 3.4 mM pNPP, and 0.01 μg/µL enzyme. Reactions were incubated at 30°C for 30 minutes and quenched with 100 mM EDTA pH 10. IC_50_ values were then calculated directly from regression curves using PRISM. All reported values are the mean of two independent experiments.

### Peptide-based assays

The phospho-peptide KKATQASQEpY was obtained from Genscript. Peptide assays were conducted in 20 mM MES pH 6.5, 25 mM NaCl, 2 mM MgCl_2_, and a range of peptide concentrations from 0 to 300 µM as previously described [2]. IC_50_ values were then calculated in PRISM.

### Docking

Compound 1 was docked into the active site of EYA3(ED) using ICM Chemist Pro (Molsoft L.L.C.) with flexible ligand and rigid target. The formal charge on the Mg ion was set to +0.66. Once the ligand was docked into the active site the side-chains lining the binding pocket were refined and the ligand re-docked. A stack of docking conformations were generated and visually assessed. The orientation of compound 1 shown in Figure 2 represents a cluster with an ICM Score  = −24.8 that was significantly better than all of the other clusters.

### Mouse Aortic ring assay **(**ARA**)**

The preparation of rat collagen and the aortic ring assay were carried out as previously described [49], [50]. Mice were 4 to 6 week old female C57BL/6, and aortic ring explants were cultured at 37°C in a humidified incubator under a 5% CO_2_ atmosphere.

Each inhibitor was serially diluted (between 0.25 mM and 7.5 mM) to stocks of 1000× the working concentrations in sterile 100% DMSO in order to achieve an equal final concentration of DMSO (0.1% v/v) in all culture conditions. For the experiments, the compounds were diluted in EBM containing 2.5% FBS, penicillin/streptomycin and 20 ng/ml VEGF165 (R&D Systems, Minneapolis, MN). Each compound was applied from the first day of culture over a 10-day period with medium change every two days. In all experiments, the vehicle (DMSO, 0.1%) was used as control. The sprouting density was derived from counting the number of branches per ring, and the extent of angiogenic sprouting was estimated by measuring the total surface area covered by the vessels.

### Analysis of zebrafish vasculature

Tg(*kdrl*:EGFP)^s843^ zebrafish line was used for vascular analysis. Embryos were maintained in fish water until the 50% epiboly stage (5.25 hpf) and were transferred into wells of 24-well plate containing the inhibitors in fish water. All incubation was performed at 28.5°C. The analysis for vascular defects was performed at the 28–30 hpf stages, and embryos were imaged using a fluorescence microscope.
